# Building Global Capacity for Conducting Operational Research Using the SORT IT Model: Where and Who?

**DOI:** 10.1371/journal.pone.0160837

**Published:** 2016-08-09

**Authors:** Rony Zachariah, Stefanie Rust, Selma Dar Berger, Nathalie Guillerm, Karen Bissell, Paul Delaunois, Anthony J. Reid, Ajay M. V. Kumar, Piero L. Olliaro, John C. Reeder, Anthony D. Harries, Andrew Ramsay

**Affiliations:** 1 Médecins Sans Frontières, Medical Department, Brussels Operational Centre, MSF-Luxembourg, Luxembourg; 2 KNCV, Tuberculosis Foundation, The Hague, Netherlands; 3 International Union Against Tuberculosis and Lung Disease, Paris, France; 4 School of Population Health, University of Auckland, Auckland, New Zealand; 5 International Union Against Tuberculosis and Lung Disease, South-East Asia Regional Office, New Delhi, India; 6 Special Programme for Research and Training in Tropical Diseases (TDR), World Health Organization, Geneva, Switzerland; 7 London School of Hygiene & Tropical Medicine, Keppel Street, London, United Kingdom; 8 School of Medicine, University of St Andrews, Fife, Scotland, United Kingdom; IRCCS Istituto Auxologico Italiano, ITALY

## Abstract

**Setting:**

Research capacity is weakest in low and middle-income countries (LMICs) where operational research is highly relevant and needed. Structured Operational Research and Training Initiative (SORT IT) courses have been developed to train participants to conduct and publish operational research and influence policy and practice. Twenty courses were completed in Asia, Africa, Europe and the South Pacific between 2009 and 2014.

**Objectives:**

In the 20 completed SORT IT courses, to assess where the research was conducted, who was trained, who became facilitators in subsequent courses and course outcomes.

**Design:**

A cohort study of completed SORT IT courses

**Results:**

There were 236 participants (41% female) including 64 nationalities who conducted research in 59 countries, mostly from Asia and Africa (mean course duration = 9.7 months). Most participants (68%) were from government health programs and non-governmental agencies. A total of 213(90%) participants completed all milestones successfully with 41(19%) becoming subsequent course facilitators, 88% of whom were from LMICs. Of 228 manuscripts submitted to scientific journals, 197(86%) were either published or in press; in 86%, the principal investigator (first author) was a LMIC national. Papers were published in 23 scientific journals (impact factor 0.5–4.4) and covered 21 disease categories (median publication time = 5.7 months). Published papers (186) had 94,794 cumulative article views/downloads. Article views/downloads for immediate open access articles were double those from closed access journals.

**Conclusion:**

The SORT IT model has been effective in training personnel to produce relevant operational research in LMICs. It merits continued commitment and support for further scale-up and development.

## Introduction

The 2013 World Health Report of the World Health Organization (WHO) was entitled “Research for Universal Health Coverage”.[1] One of its key messages was that universal health coverage, with access to high-quality services, cannot be achieved without evidence from research.[1] Importantly, it emphasized the need to strengthen research capacity in public health programs, close to the supply and demand of health services.[1, 2]

Research capacity, is lowest in Low- and Middle-Income Countries (LMICs) where research evidence is highly relevant and most needed.[3, 4] Meeting this challenge requires developing and implementing effective models for capacity building. [2, 5, 6] One such model is the Structured Operational Research and Training Initiative (SORT IT)–a global partnership led by the Special Program for Research and Training in Tropical Diseases at WHO (WHO/TDR). SORT IT supports countries to conduct operational research around their own priorities, to build improved and sustainable research capacity, and to make evidence-informed changes in the delivery of health services. [7]

The SORT IT training courses impart the practical skills of conducting and publishing research in order to influence policy and practice.[8] There is an emphasis on publishing in peer-reviewed scientific journals as an indicator of successful completion of planned research [2] and a scientific quality control standard. Publication enhances the credibility of research findings,[9] and provides robust documentation of evidence for policy review. Measuring the real impact of research is a long-term effort, however, the published paper is an important milestone in the journey to achieving impact on the ground. [10]

Between 2009 and 2014, 20 SORT IT courses were completed world-wide. Previous studies on these courses have reported high publication yields and self-reported influence on policy and practice.[11–14] However, to date, there has been no detailed assessment of “where” this research has been done “who” has been trained and who amongst those trained became facilitators. This information would be useful to assess patterns in the capacity being built by SORT IT.

Furthermore, assessing whether research evidence is available in a timely and “openly accessible” manner is important for operational research. [10, 15, 16] Internet downloads and article views are useful parameters with which to judge research access and this assessment has yet to be done.

We aimed to describe SORT IT participant profiles, course outcomes and characteristics of published papers. Specific objectives were to report on the:

1. geographic location of the research projects, the socio-demographic profiles of participants, their nationalities and institutional affiliations.
2. number and profile of participants who successfully completed the course, published their research and became facilitators on subsequent courses.
3. characteristics of publications, including time to publication, immediate open access publication costs, and article views and downloads.

## Methods

### Design and participants

This was a retrospective cohort study involving all participants (and outcomes) of 20 completed SORT IT courses conducted in Europe (France, Luxembourg and Estonia), Asia (India and Nepal), Africa (Kenya and Ethiopia) and the South Pacific (Fiji). In the courses conducted in France and Luxembourg, most participants were nationals from Africa or Asia. Other courses chose participants from their respective region or country. All reported courses were conducted between 2009 and 2014.

### Participant profiles

All applicants submitted a detailed application containing their socio-demographic information, nationality, project location, institutional affiliations, field of work and ideas for a research project. A maximum of 12 candidates, who fulfilled strict selection criteria, were enrolled per course[8]. These criteria were designed to maximize individual and institutional commitment to course completion. Most candidates, who were chosen, were new to research.[17]

### SORT IT and criteria for success

The details of SORT IT courses, including participant selection criteria, milestones, mentorship and the metrics of assessment have been described previously.[8] In brief, the course was comprised of three modules conducted over a period of 10 to 12 months: Module 1 (5–6 days) focused on development of a study protocol, leading to Module 2 (5–6 days) on efficient data capture and analysis. Module 3 (5–7 days) focused on writing a manuscript for scientific publication.[10] Specific milestones needed to be achieved if participants were to proceed from one module to the next. A participant was judged to have successfully completed the course if s/he completed all milestones, including submission of a completed manuscript to a peer-reviewed journal within four weeks of completing Module 3. Milestone achievement was closely monitored by course coordinators.

Successful course participants, who showed high motivation, active participation and quick learning skills as judged by senior facilitators at each course, were invited to participate in future courses as facilitators. A dedicated SORT IT facilitator inventory was used to compile data on the numbers of former participants who became facilitators on subsequent SORT IT courses.

### Publication outputs and characteristics

Publication outputs were assessed and reported by course coordinators on a quarterly basis and they included original articles and viewpoint articles. Viewpoint articles stemming directly from iterative discussions within the courses and that challenged “business as usual” were included. For manuscripts, the date of initial and subsequent submission to any journal, name of the journal and eventual date of publication were recorded. This allowed calculation of “time to publication”.

Information on whether a given publication was immediate open access, closed or delayed open access was sought from the journal website on the censor date. Immediate open access publishing costs were based on journal rates.

The number of publication downloads were sourced from the journal websites or Ingenta publishing (if an Ingenta journal). Citation counts were retrieved from Google Scholar. Cumulative publication outputs were assessed for the 20 courses, which were completed between March 1^st^ 2010 and December 30^th^ 2014 and censored on 1st July 2015.

### Data collection and analysis

Information on socio-demographic characteristics of participants, course outcomes, publication outputs and new facilitators were obtained from participant application forms and/or course files. EpiData software was used for data entry (version 3.1 EpiData Association, Odense, Denmark) and STATA (Version 11, CDC, Atlanta) for analysis (S1 Variables, S1 Dataset). Maps were generated using the StatPlanet software, (http://www.statsilk.com/license) which is available free-of-charge for non-commercial use.

### Ethics

The study met the Médecins Sans Frontières Ethics Review Board (Geneva, Switzerland) approved criteria for studies of routinely collected data. The Ethics Advisory Group of the International Union Against Tuberculosis and Lung Disease (Paris, France) also exempted the study from ethics review. This study was a review of records and involved anonymized and de-identified data prior to analysis. Hence, the need for individual informed consent was waived by the named ethics committees.

## Results

### Geographical location and research projects

There were a total of 20 completed SORT IT courses lasting between seven and 12 months (mean = 9.7 months) between protocol development and manuscript completion. Course locations were Europe -Paris (4), Luxembourg (3), Tallinn (1); Africa-Nairobi (1) and Addis Ababa; (2), Asia- Kathmandu (3), Chennai (2), Hyderabad (1); and South Pacific- Fiji (3). A total of 236 participants were enrolled with research projects from 59 countries.

**Fig 1:**
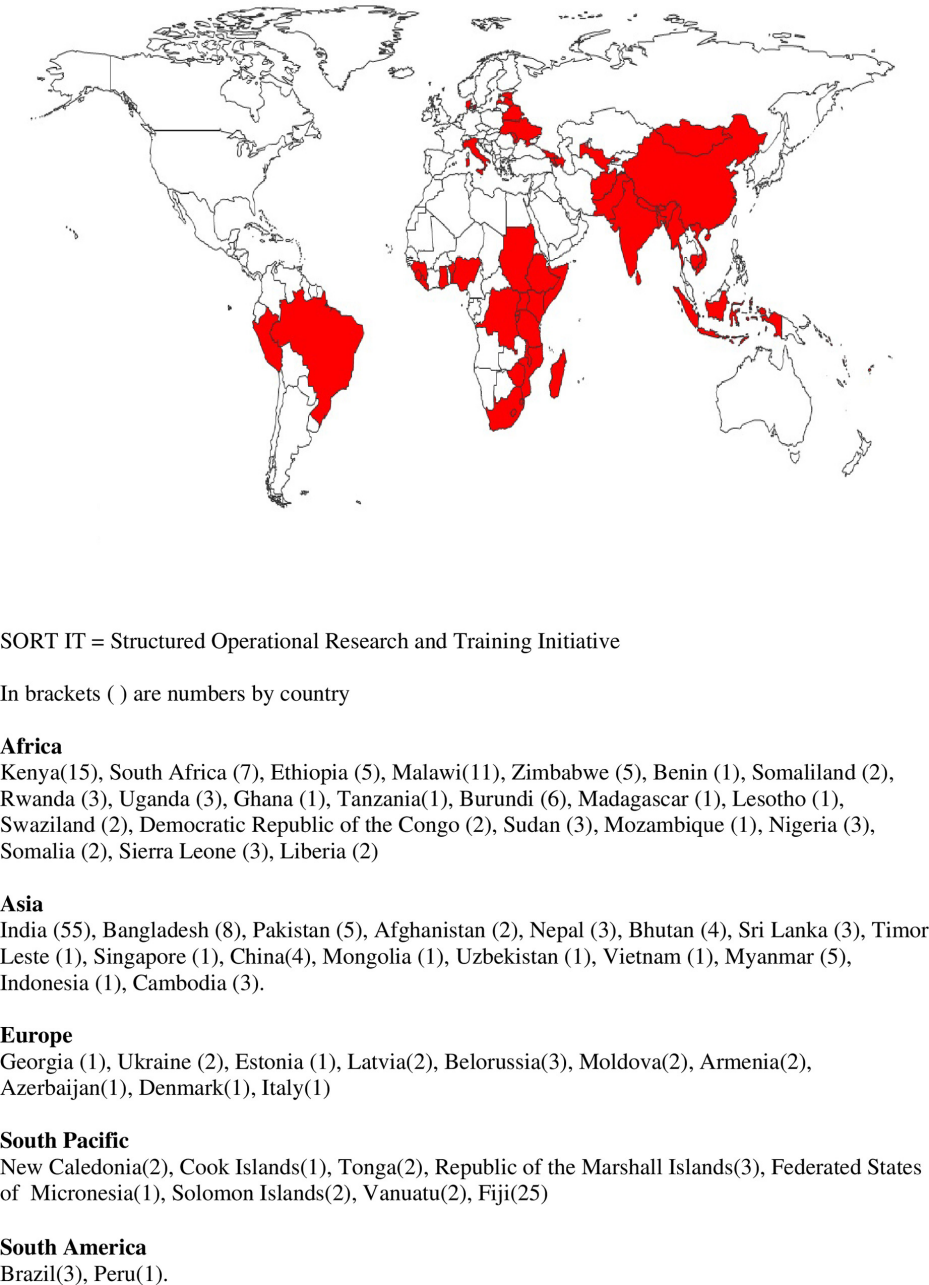
SORT IT Operational Research capacity building projects involving 236 participants from 59 countries (2009–2014).

**Fig 1**shows the location of SORT IT operational research projects. Of note, research projects were conducted in 59 countries, the great majority located in LMICs. [18]

### Socio-demographic characteristics and institutional affiliations

Of 236 participants (41% female, age 27–61 years), there were 64 nationalities including: 19 countries from Africa, 16 from Asia, nine from Eastern Europe, eight from the North and South Pacific, seven from Western Europe, three from Latin America, and one each from USA and Australia respectively. Individuals with nationalities from Western Europe, USA and Australia were all from institutional headquarters and directly involved with research projects in LMICs.

**Table 1**shows the sociodemographic profile, work sites and institutional affiliations of participants. Most (68%) were medical care providers (doctors, nurses or clinical officers) working within public health programs of Ministries of Health (MOH) or with non-governmental organizations (NGOs). Occupations outside the health sector included education, agriculture, and social welfare. Participants working with vulnerable populations comprised 5% of the cohort.

**Table 1:** Socio-demographic characteristics and institutional affiliations of participants in 20 completed courses of the Structured Operational Research and Training Initiative (SORT IT, 2009–2014).

Characteristic		N	(%)
**Total**		236	
**Median age (range)**		37 (29–61)	-
**Gender**			
	Male	140	(59)
	Female	96	(41)
**Occupation**			
	Medical doctor, Nurse, Clinical officer	158	(68)
	Research officer/Epidemiologist/Data manager	39	(17)
	Public health officer	18	(8)
	Pharmacist	6	(3)
	Laboratory technician	6	(3)
	Lecturer	3	(1)
	Nutritionist	3	(1)
	Agricultural officer	1	(<1)
	Teacher	1	(<1)
	Social scientist	1	(<1)
**Affiliated institutions**			
	Ministry of Health/public health programs	115	(49)
	International or national NGOs	77	(32)
	Academic institutions	42	(18)
	Other–WHO/Donors	2	(1)
**Work site**			
	Institutional headquarters	127	(54)
	District or health facility	88	(37)
	Vulnersable population groups*	11	(5)
	Community	10	(4)

NGO–non-governmental organization

*Includes most at-risk populations such as commercial sex workers, intravenous drug users, and prisoners

SORT IT = Structured Operational Research and Training Initiative.

### Course completion, publication outputs and subsequent facilitation by participants

**Fig 2:**
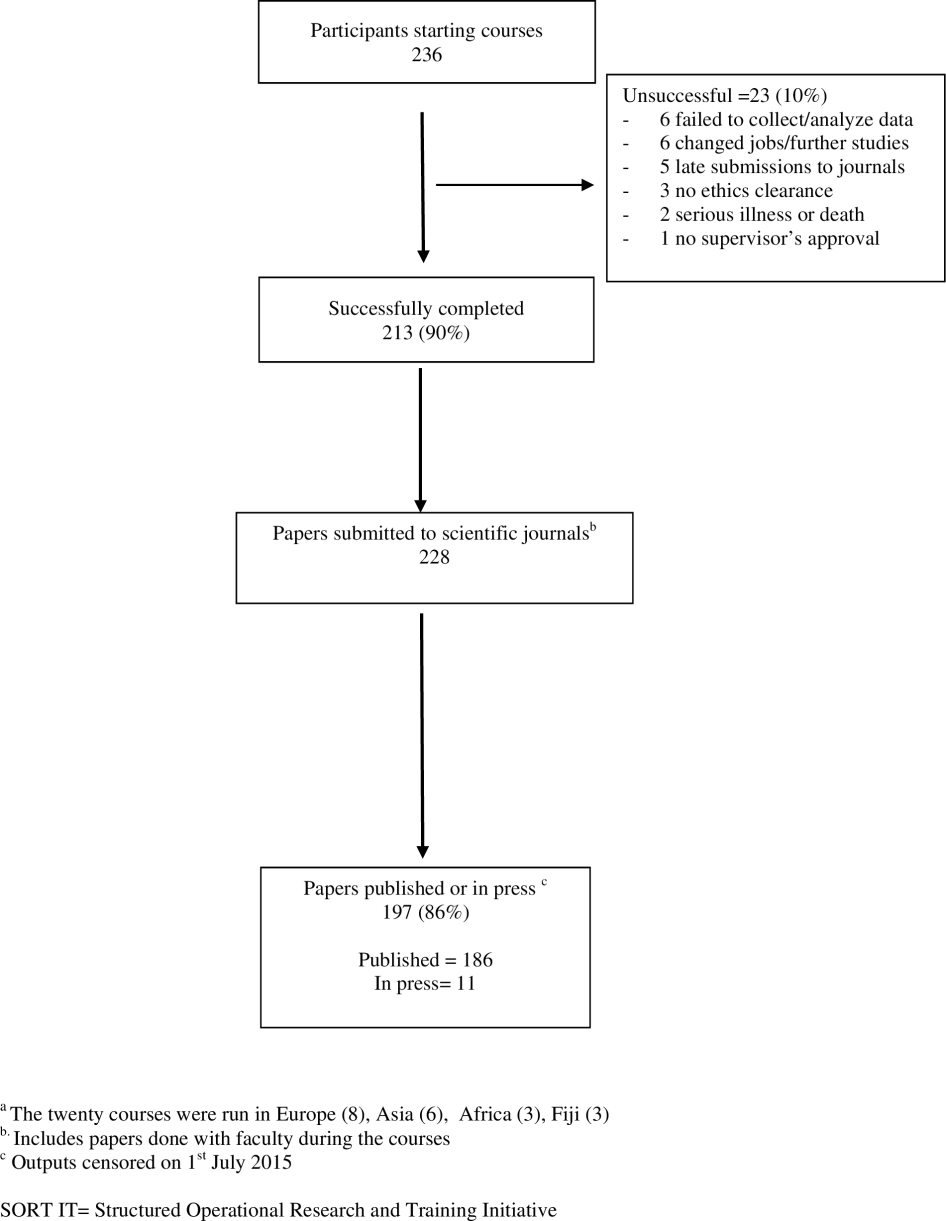
Course completion and publication outputs from 20 completed SORT IT courses in Europe, Africa, Asia and the South Pacific, (2009 to 2014).

Course completion and publication outputs are shown in **Fig 2.** Of 236 participants, 213 (90%) completed all milestones (“successful course completion”). There were 23 (10%) participants who did not complete the course and were considered “unsuccessful”. The main reasons for this unfavorable outcome included: failure to collect or analyze data (6); changed jobs or left for further studies abroad (6); did not complete a paper submission (5); and did not obtain ethics clearance (3) **(Fig 2)**.

A total of 228 manuscripts were submitted to peer-reviewed scientific journals (**Fig 2**). Of these, 197 (86%) were either published (186) or in press (11) on the censor date.

Of the 213 participants who achieved successful course completion, 41(19%) became new facilitators on subsequent courses (mean number of modules facilitated = 5.7, range 1–29). The average number of facilitators generated per course was two. **Table 2**shows the characteristics of these new facilitators. Most (88%) were from LMICs and 37% were female. The most frequent institutional affiliations were Ministry of Health (37%) and International NGOs (34%). Participant facilitators comprised 25 nationalities including 16(39%) from Asia, 10 (24%) from Africa, five (12%) from East Europe, four (10%) from West Europe and two (5%) each from the Pacific and USA and one each from Latin America and Australia.

**Table 2:** Characteristics of participants who became facilitators from 20 completed courses of the Structured Operational Research and Training Initiative (SORT IT, 2009–2014).

Characteristic		N	(%)
**Total number of participants (n-236) who became facilitators**		41	
**Gender**			
	Female	15	(37)
	Male	26	(63)
**Institutional affiliation**			
	Ministry of health / Public health programs	15	(37)
	International NGO	14	(34)
	National academic institution	3	(20)
	National NGO/National foundations	8	(7)
	WHO	1	(2)
**Occupation**			
	Doctor	24	(58)
	Data manager	6	(15)
	Research officer	6	(15)
	Epidemiologist	2	(5)
	Nurse	2	(5)
	Nutritionist	1	(2)
**Work site**			
	Capital	23	(56)
	District	15	(37)
	Vulnerable populations	2	(5)
	Community	1	(2)
**Region of origin**^**a**^			
	From LMIC	36	(88)
	Others	5	(12)

NGO: Non Governmental Organisation; WHO: World Health Organisation; LMIC—Low and Middle Income Countries.

a. Numbers by region: Asia (16), Africa (10), East Europe (5), West Europe (4), Pacific (2), USA (2), Latin America (1), Australia (1)

Of new facilitators, 14 (34%) were from the Union and MSF and they undertook 127 (57%) of 224 subsequent facilitations. Thirteen (nine from LMICs) of the Union-MSF facilitators were operational research fellows, who are obliged (as part of their terms of reference) to be involved in research teaching.

### Characteristics of published papers

A total of 186 papers were published in 23 peer-reviewed scientific journals after a median time of 5.7 months from first submission (Inter-quartile range = 4.3–7.7, range 1–46 months).

The six most common journals were *Public Health Action* (PHA, 58%), *PLoS ONE (*16%), *Transactions of the Royal Society of Tropical Medicine and Hygiene* (5%), *International Journal of Tuberculosis and Lung Disease* (4%) and *Biomed Central Journals* (4%). For the 22 of the 23 indexed journals (*PHA* was not yet but scheduled to be indexed at the time of analysis), journal impact factors ranged from 0.5 to 4.4.

**Table 3**shows the characteristics of published papers. In 160 (86%) of 186 publications, the principal investigator (and first author) was from a LMIC. Publications covered 21 disease categories, the most common being tuberculosis (TB) and HIV/AIDS.

**Table 3:** Characteristics of publications from 20 completed courses of the Structured Operational Research and Training Initiative (SORT IT, 2009–2014).

Characteristic		N	(%)
**Total**		186	
**Article type**			
	Original article	174	(94)
	Perspective/Debate	12	(6)
**Principal investigator from LMIC**			
	Yes	160	(86)
	No	26	(14)
**Journal submissions**			
	1 submission prior to publication	161	(87)
	2 submissions prior to publication	25	(13)
**Access type** ^**a**^			
	Fully open access	155	(83)
	Closed	24	(13)
	Delayed open access	4	(2)
	Institutional repository access	3	(2)
**Disease category** ^**b**^			
	Tuberculosis	87	(46)
	HIV/AIDS	18	(10)
	Implementation research	15	(8)
	Bacterial diseases	8	(4)
	Non-communicable disease	8	(4)
	Nutrition	6	(3)
	Health care access	6	(3)
	Pediatrics	6	(3)
	Maternal health	6	(3)
	Smoking	4	(2)
	Antibiotic resistance	3	(2)
	Malaria	3	(2)
	Cancers	3	(2)
	Knowledge management	3	(2)
	Sexual violence	2	(1)
	Leishmaniasis	2	(1)
	Research capacity strengthening	2	(1)
	Surgery	1	(1)
	Lassa fever	1	(1)
	Research tools	1	(1)
	Research ethics	1	(1)

a. Definitions as per reference 15

b. Categorization as recommended by The Special Programme for Research and Training in Tropical Diseases at the World Health Organization (WHO/TDR), Geneva, Switzerland

SORT IT = Structured Operational Research and Training Initiative.

### Internet article views, downloads and citations

Published papers (n-186) had a cumulative total of 74,802 article views (mean = 402, range 0–5512), a cumulative total of 19,992 downloads (mean = 107, range 0–1092), and 504 citations.

Of 155 (82%) open access publications, there were 87,810 (563 per published paper) cumulative article views and downloads compared to 6,402 for 24 closed access publications (267 per published paper).

### Open access costs

The total cost of 155 immediate open access publications was 226,115 Euros (average cost per paper = 1,459 Euros, range = 150–4500) (**Table 3**). The least expensive journal was the *Pan African Medical Journal* while the most expensive was *Lancet Global Health*.

**Fig 3:**
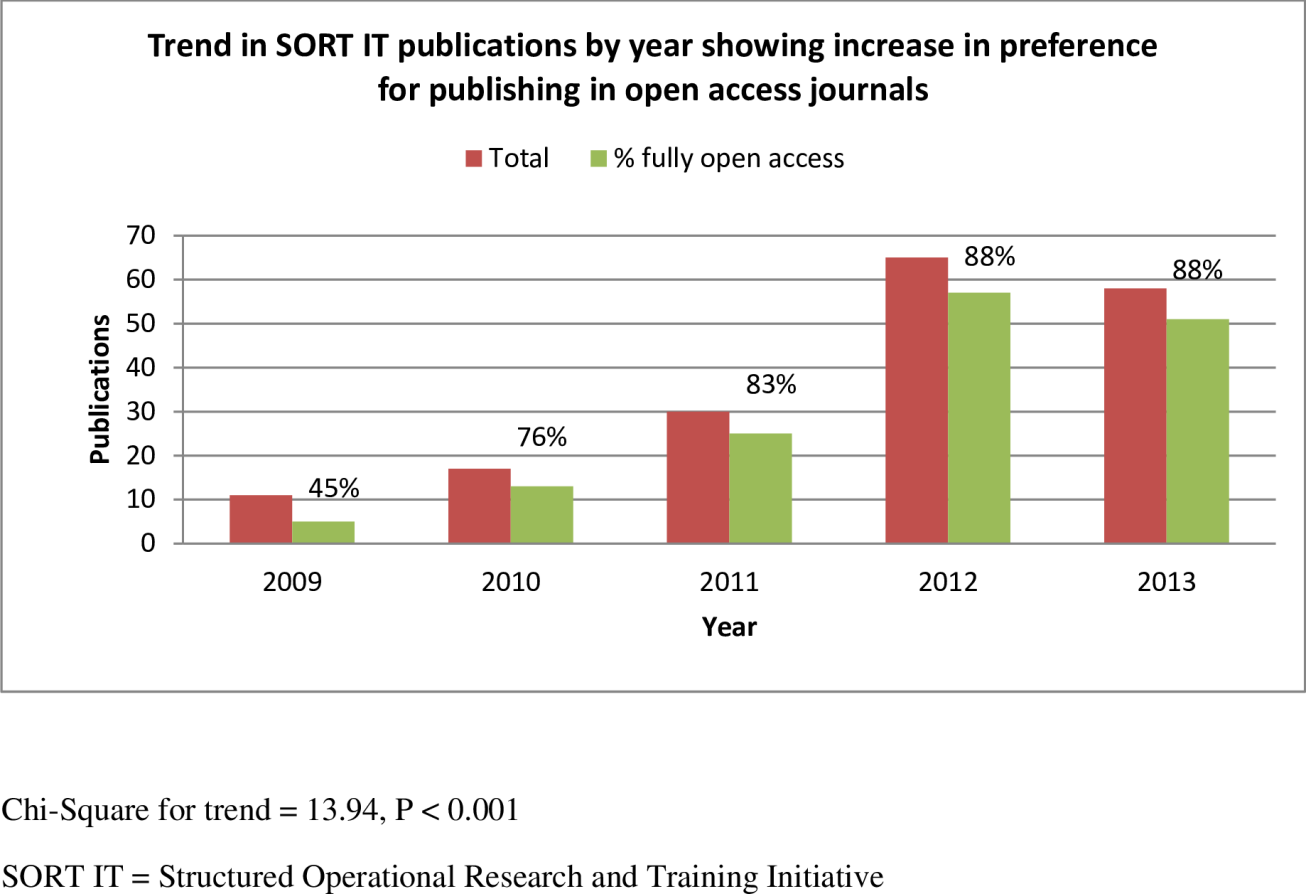
Trend in number of publications from SORT IT courses and the proportion of open access journal publications (2009–2013).

**Fig 3**shows the trend in open access publications for the period 2009 to 2013 (2014 was excluded as there were only four publications at censor date). A progressive and linear trend was noted, reaching ≥80% in later years. (Chi-square for trend = 13.94, *P*<0.001)

## Discussion

This is the first study to assess the “where” and “who” of the SORT IT global capacity building initiative in operational research. Most participants were from a wide range of LMICs, and almost one-in-five become facilitators in subsequent courses. There were high publication outputs, in a timely manner, with the majority in immediate open access journals.

These findings are of importance for two reasons. First, a few years ago, Ian Chalmers and Paul Glasziou, estimated that close to 85% of all research investment was being wasted.[19, 20] Their message was that medical research is wasteful if it is not published and if it does not contribute to improvements in health care delivery. As the SORT IT program demands research completion and publication, it reduces research waste. Furthermore, two other studies have already shown that between 55% to 74% of SORT IT publications had an influence on policy and/or practice. [12, 21] Second, SORT IT focuses on participants from (or directly involved with) LMICs, where the greatest research gaps exist and where it is vital to improve research capacity. [18]

The study strengths are that information on all participants, their milestone achievements and publication outputs were carefully archived, allowing rigorous tracking of participants and publication outputs. A limitation is that the follow-up time was different for the various courses and we may have underestimated the publication rate and the number of article views and downloads. Of note, the Journal, *Public Health Action*, was not accessible through PubMed at the time of censoring, and being the choice for over half of all published papers, its absence may have underestimated the reported article views and downloads. In addition, we only recorded facilitation on SORT IT courses and may have not counted contributions to other courses, thereby underestimated this parameter.

This study reports a number of important findings. First, the great majority of participants were from public health programs of MoH or NGOs, which represents a step towards implementers embracing research as part of their work. Furthermore, roughly one-in-five participants became facilitators on subsequent SORT IT courses, thereby demonstrating improved research and training capacity. This finding is further endorsed by two other studies, [11, 22] showing that research output continued well beyond SORT IT course completion with about 50% of participants completing new research studies and 40% getting published.

Second, although the majority of participants were from the traditional health sector, there were a few participants that worked with vulnerable and often excluded groups such as commercial sex workers, and drug users. As well, there was one participant each from non-health sectors (education, agriculture and social science) which is encouraging. In addition, four in ten successful participants were women and this is important in terms of gender equity. Streamlining the SORT IT “way of learning” to mesh with the new Sustainable Development Goals (SDGs) may help expand the use of the SORT IT model to other domains. [23] The fact that publications covered a wide spectrum of disease categories suggests that the approach could be applied to a wider range of health systems.

Third, the average time from first submission to publication was less than six months, which is timely for making relevant evidence available to public health programs. The relatively short time period is due to two principal factors–choice of journals which have quicker turn-around times and are supportive of operational research and sustained support by experienced mentors during the peer-review process and re-submission. The fact that the majority of publications were in categories of TB and HIV/AIDS is also related to data availability–both disease control programs have structured quarterly reporting and monitoring systems which facilitate operational research. As individuals and countries evolve in their basic capacity to conduct and publish operational research, the need to diversify the portfolio of disease categories and take on more sophisticated research designs is evident.

Fourth, although it is encouraging that about one in five participants became facilitators, there is no yardstick with which to compare this outcome. A proportion of almost 20% becoming facilitators is not unreasonable given the intense nature of the course and the need for facilitators to commit for several months (or years) until publication, SORT IT is unlike other training courses where facilitators come for a week or two, teach and then leave “hands-free” with no post-course mentorship commitment. SORT IT facilitators need to have a combination of several qualities including technical competence, an interest for teaching and high motivation for hard and largely pro-bono work. Importantly, most course participants came from busy and overworked public health programs and they already find difficulty getting dedicated time away from their usual workload to complete their SORT IT course requirements. Thus, they may have even less opportunity to become facilitators. Another factor hindering availability may be socio-cultural factors preventing some female facilitators from travelling to distant course locations. Therefore, “interest and competence” in facilitation may not necessarily translate into “ability” and “willingness” to do so. We know of several participants who were competent to facilitate, but were unable or unwilling to commit to this role.

A considerable proportion of all mentoring was done by facilitators from two NGOs (The Union and MSF) that were pioneers in its development and have continued their institutional commitment to SORT IT. These two institutions have introduced innovative operational research fellowships which included facilitation as part of individual capacity development.

With roughly two participants being identified as potential facilitators on each SORT IT course, it will take about three to four years for a country to reach the current minimum SORT IT requirement of 6–8 facilitators for 10–12 participants. Possible ways for generating new SORT IT facilitators may include: formulating objective criteria for identifying potential facilitators on all courses; pairing senior and junior facilitators to enhance on-the-job learning; building facilitator pools around countries or regional courses; negotiating with institutions to allow dedicated time for facilitation; providing nominal honorariums; and extending the operational research fellowship program to public health programs. We also need to identify and bring in experienced operational researchers with good understanding of public health programs from outside the SORT IT network to facilitate at country level.

Finally, in order to foster dissemination of operational research studies, and to meet WHO requirements, we chose “open access” journals for publication wherever possible. The benefit of this approach is that it fosters access and dissemination of published evidence in LMICs, without cost to readers. A significant down side is the high cost for open access which highlights the need for better planning and dedicated funding.[16]

In conclusion, in a wide range of LMICs, where operational research is much needed, the SORT IT model contributed to building research capacity and produced relevant evidence. There was a very high publication rate. Most participants were from LMICs, 40% were women, and their studies were focused on programmatic issues; almost 20% went on to become facilitators in other courses. The SORT IT model deserves to be taken up and expanded further to address the urgent health needs at program level and contribute towards achieving universal health coverage.

## Supporting Information

S1 Dataset Dataset for SORT IT courses. (XLSX)

S1 Variables Documentation sheet. Variables and codes.
(DOCX)
