# Supplementary material for: Building Global Capacity for Conducting Operational Research Using the SORT IT Model: Where and Who?
Source: PLoS One. 2016 Aug 9;11(8):e0160837. doi: 10.1371/journal.pone.0160837 (PMC4978462; doi:10.1371/journal.pone.0160837)
Supplement: S1 Variables — Variables and codes. (DOCX) [file pone.0160837.s002.docx]

**Data documentation sheet for SORT IT study – SORT IT capacity building in operational research: who and where?**

| **Field name** | **Field label** | **Field type** | | **Field length** | **Field values** | **Value labels** | | **Comments** |
| --- | --- | --- | --- | --- | --- | --- | --- | --- |
| **Objective 1: The socio-demographic profile of participants** | | | | | | | |  |
| ID | Participant id | | Numeric | 3 |  |  | |  |
| Course | Title of course | | Numeric | 2 | 1  2  3  4  5  6  7  8  9  10  11  12  13  14  15  16  17  18  19  20 | Paris 1  PHFI 1  Paris 2  Paris 3  Lux 1  Fiji 1  Asia 1  Africa 1  Chennai 1  Lux 2  Paris 4  SP 1  Asia 2  Africa 2  Fiji 2  Estonia 1  Lux 3  Chennai 2  Asia 3  Africa 3 | | Completed courses in chronological order |
| Start | Course start date | | Date | 10 | 03/08/2009-21/11/2014 |  | | Range of legal dates |
| End | Course end date | | Date | 10 | 05/03/2010-28/11/2015 |  | | Range of legal dates |
| duration | Course duration | | Numeric | 4 |  |  | | number of months |
| Location | Course location | | Numeric | 2 | 1  2  3  4  5  6  7  8  9  10  11 | Paris  Hyderabad  Luxembourg  Fiji  Kathmandu  Nairobi  Chennai  Addis Ababa  Tallinn  Astana  Panama | | Name of city |
| Name | Participant name | | Text | 25 |  |  | |  |
| Age | Age in years | | Numeric | 2 | 1-80  99 | Not recorded | |  |
| Sex | Persons gender | | Numeric | 1 | 1  2  9 | Male  Female  Not recorded | |  |
| Country | Project country | | Numeric | 2 | 1  2  3  4  5  6  7  8  9  10  11  12  13  14  15  16  17  18  19  20  21  22  23  23  25  26  27  28  29  30  31  32  33  34  35  36  37  38  39  40  41  42  43  44  45  46  47  48  49  50  51  52  53  54  55  56  57  58  59  60  61  62  63  64  65  66  67  68  69  70  71  72  73 | Georgia  Ukraine  Estonia  Latvia  Belorussia  Moldova  Armenia  Azerbaijan  Denmark  Italy  Switzerland  Brazil  Peru  Mexico  El Salvador  Columbia  Dominican Rep  Guatemala  Hondurus  Haiti  Kenya  South Africa  Ethiopia  Malawi  Zimbabwe  Benin  Somaliland  Rwanda  Uganda  Ghana  Tanzania  Burundi  Madagascar  Lesotho  Swaziland  DRC  Sudan  Mozambique  Nigeria  Somalia  Sierra leone  Liberia  Guinea  Ivory Coast  India  Bangladesh  Pakistan  Afghanistan  Nepal  Bhutan  Sri-Lanka  Uzbekistan  Tadjikistan  Singapore  China  Vietnam  Mongolia  Cambodia  Myanmar  Kazaksthan  Turkmesistan  Kyrgysthan  Timor Leste  Indonesia  New Caledonia  Cook Islands  Tonga  Marshal Islands  Micronesia  Solomon Island  Vanuatu  Fiji  na | |  |
| Occup | Participants occupation | | Numeric | 2 | 1  2  3  4  5  6  7  8  9  10  11  12  13  14  15  16 | Medical Doctor  Dentist  Clinical officer  Nurses  Data managers  Epidemiologist  Research officer  Lab technician  Nutritionist  Pharmacist  Public health officer  Teacher  Social scientist  Health economist  Agriculturist  Academic | |  |
| Instit | Affiliated institution | | Numeric | 1 | 1  2  3  4  5  6  7 | MOH  National program  International NGO  National NGO  National Acad instiit  International Acad Instit  Other | |  |
| Field | Field/disease of work | | Numeric | 2 | 1  2  3  4  5  6  7  8  9  10  11  12  13  14  15  16  17 | Operations research  Community  Health systems  Sexual violence  Paediatrics  Maternal health  Mental health  Surgery  Pharmacy  TB  HIV/AIDS  Malaria  Neglected diseases  Nutrition  NCDs  Cancers  Others | |  |
| Site | Work site | | Numeric | 1 | 1  2  3  4  5  6 | HQ/capital  District  Health facility  Community  Vulnerable populations  Others | |  |
| **Objective 2/ 3 Number who complete and publish their research and characteristics of publications** | | | | | | | | |
| Outcome | Course outcome | | Numeric | 1 | 1  2 | Success  Failure | |  |
| Failure | Reason for course failure | | Numeric | 1 | 1  2  3  4  5  6  7  8  9 | No supervisors OK  No ethics  Changed jobs  Serious illness  No data analysis done  No data collected  Delayed submission  Died  Started MpH | |  |
| Nopapers | No of papers(P) submitted | | Numeric | 1 | 1-4 |  | |  |
| SubdateP1 | Date of first journal submissionP1 | | Date | 10 | DD/MM/YYYY |  | |  |
| TotalSubP1 | Total number of submissionsP1 | | Numeric | 2 | 1-10 |  | |  |
| StatusP1 | Manuscript status at date of censurP1 | | Numeric | 1 | 1  2  3  4 | Submitted  In press  Published  Dropped | | Dropped=given up |
| PubdateP1 | Date of publicationP1 | | Date | 10 | DD/MM/YYYY |  | |  |
| PubnameP1 | Journal where publishedP1 | | Numeric | 2 | 1  2  3  4  5  6  7  8  9  10  11  12  13  14  15  16  17  18  19  20  21  22 | | IJTLD  TMIH  TRSTH  PlosOne  BMC Infec Dis  BMC ResNotes  BMC Pub Health  BMC H Serv  PHA  Health Ed J  Int Health  Current Scien  J Trop Med  JIAS  JAIDS  GHA  Int Soc Sex Med  Pan Afr Med J  NZMJ  Lancet  Am J Inf Cont  J Trop Ped |  |
| PubtypeP1 | Article typeP1 | | Numeric | 1 | 1  2 | | Original research  Viewpoint |  |
| DiscatP1 | Disease categoryP1 | | Numeric | 2 | 1  2  3  4  5  6  7  8  9  10  11  12  13  14  15  16  17 | | Bacterial diseases  TB  Leprosy  Malaria  Leishmaniasis  Trpanosomiasis  Chagas disease  Onchocerhiasis  Lymphatic filariasis  Schistomosomiasis  Other helminths  Dengue  HIV/AIDS  Lassa fever  NCDs  Nutrition  cancers |  |
| PIP1 | PI from Low income countryP1 | | Numeric | 1 | 1  2 | | Yes  No |  |
| AccessP1 | Access typeP1 | | Numeric | 1 | 1  2  3  4 | | Journal- Immed open access  Journal.-delayed open access  Repository open access  Closed |  |
| CostP1 | Cost if open accessP1 | | Numeric | 4 | 0-7000 | | Cost in USD |  |
| JIFP1 | Journal impact factorP1 | | Numeric | 4 | 1.0-10.0  99 | | Range of legal values  Unknown/not yet on pubmed |  |
| WHOP1 | Copyright of WHOP1 | | Numeric | 1 | 1  2 | | Yes  No |  |
| Artview | Article views | | Numeric | 4 |  | |  |  |
| artdownload | Article downloads | | Numeric | 4 |  | |  |  |
| gogscholcit | Google scholar citations | | Numeric | 4 |  | |  |  |
| DInjentaP1 | Injenta downloadsP1 | | Numeric | 4 | 0-9000 | |  | Range of legal values |
| SubdateP2 | Date of first journal submissionP2 | | Date | 10 | DD/MM/YYYY |  | |  |
| TotalSubP2 | Total number of submissionsP2 | | Numeric | 2 | 1-10 |  | |  |
| StatusP2 | Manuscript status at date of censurP2 | | Numeric | 1 | 1  2  3  4 | Submitted  In press  Published  Dropped | | Dropped=given up |
| PubdateP2 | Date of publicationP2 | | Date | 10 | DD/MM/YYYY |  | |  |
| PubnameP2 | Journal where publishedP2 | | Numeric | 2 | 1  2  3  4  5  6  7  8  9  10  11  12  13  14  15  16  17  18  19  20  21  22 | | IJTLD  TMIH  TRSTH  PlosOne  BMC Infec Dis  BMC ResNotes  BMC Pub Health  BMC H Serv  PHA  Health Ed J  Int Health  Current Scien  J Trop Med  JIAS  JAIDS  GHA  Int Soc Sex Med  Pan Afr Med J  NZMJ  Lancet  Am J Inf Cont  J Trop Ped |  |
| PubtypeP2 | Article typeP1 | | Numeric | 1 | 1  2 | | Original research  Viewpoint |  |
| DiscatP2 | Disease categoryP1 | | Numeric | 2 | 1  2  3  4  5  6  7  8  9  10  11  12  13  14  15  16  17 | | Bacterial diseases  TB  Leprosy  Malaria  Leishmaniasis  Trpanosomiasis  Chagas disease  Onchocerhiasis  Lymphatic filariasis  Schistomosomiasis  Other helminths  Dengue  HIV/AIDS  Lassa fever  NCDs  Nutrition  cancers |  |
| PIP2 | PI from Low income countryP2 | | Numeric | 1 | 1  2 | | Yes  No |  |
| AccessP2 | Access typeP2 | | Numeric | 1 | 1  2  3  4 | | Journal- Immed open access  Journal.-delayed open access  Repository open access  Closed |  |
| CostP2 | Cost if open accessP2 | | Numeric | 4 | 0-7000 | | Cost in USD |  |
| JIFP2 | Journal impact factorP2 | | Numeric | 4 | 1.0-10.0  99 | | Range of legal values  Unknown/not yet on pubmed |  |
| WHOP2 | Copyright of WHOP2 | | Numeric | 1 | 1  2 | | Yes  No |  |
| Artview | Article views | | Numeric | 4 |  | |  |  |
| artdownload | Article downloads | | Numeric | 4 |  | |  |  |
| Gogscholcit | Google scholar citations | | Numeric | 4 |  | |  |  |
| DInjentaP2 | Injenta downloadsP2 | | Numeric | 4 | 0-9000 | |  | Range of legal values |
| DPubmedP2 | Pubmed downloadsPé | | Numeric | 4 | 0-9000 | |  | Idem |
| SubdateP3 | Date of first journal submissionP3 | | Date | 10 | DD/MM/YYYY |  | |  |
| TotalSubP3 | Total number of submissionsP2 | | Numeric | 2 | 1-10 |  | |  |
| StatusP3 | Manuscript status at date of censurP3 | | Numeric | 1 | 1  2  3  4 | Submitted  In press  Published  Dropped | | Dropped=given up |
| PubdateP3 | Date of publicationP3 | | Date | 10 | DD/MM/YYYY |  | |  |
| PubnameP3 | Journal where published3 | | Numeric | 2 | 1  2  3  4  5  6  7  8  9  10  11  12  13  14  15  16  17  18  19  20  21  22 | | IJTLD  TMIH  TRSTH  PlosOne  BMC Infec Dis  BMC ResNotes  BMC Pub Health  BMC H Serv  PHA  Health Ed J  Int Health  Current Scien  J Trop Med  JIAS  JAIDS  GHA  Int Soc Sex Med  Pan Afr Med J  NZMJ  Lancet  Am J Inf Cont  J Trop Ped |  |
| PubtypeP3 | Article typeP3 | | Numeric | 1 | 1  2 | | Original research  Viewpoint |  |
| DiscatP3 | Disease category3 | | Numeric | 2 | 1  2  3  4  5  6  7  8  9  10  11  12  13  14  15  16  17 | | Bacterial diseases  TB  Leprosy  Malaria  Leishmaniasis  Trpanosomiasis  Chagas disease  Onchocerhiasis  Lymphatic filariasis  Schistomosomiasis  Other helminths  Dengue  HIV/AIDS  Lassa fever  NCDs  Nutrition  cancers |  |
| PIP3 | PI from Low income countryP3 | | Numeric | 1 | 1  2 | | Yes  No |  |
| AccessP3 | Access typeP3 | | Numeric | 1 | 1  2  3  4 | | Journal- Immed open access  Journal.-delayed open access  Repository open access  Closed |  |
| CostP3 | Cost if open accessP3 | | Numeric | 4 | 0-7000 | | Cost in USD |  |
| JIFP3 | Journal impact factorP3 | | Numeric | 4 | 1.0-10.0  99 | | Range of legal values  Unknown/not yet on pubmed |  |
| WHOP3 | Copyright of WHOP3 | | Numeric | 1 | 1  2 | | Yes  No |  |
| Artview | Article views | | Numeric | 4 |  | |  |  |
| artdownload | Article downloads | | Numeric | 4 |  | |  |  |
| Gogscholcit | Google scholar citations | | Numeric | 4 |  | |  |  |
| DInjentaP3 | Injenta downloadsP3 | | Numeric | 4 | 0-9000 | |  | Range of legal values |
| DPubmedP3 | Pubmed downloadsP3 | | Numeric | 4 | 0-9000 | |  | Idem |
| DMSFP3 | MSF downloads P3 | | Numeric | 4 | 0-9000 | |  | Idem |
| DJournalP3 | Journal site downloadP3 | | Numeric | 4 | 0-9000 | |  | Idem |
| CInjentaP3 | Injenta citationsP3 | | Numeric | 4 | 0-9000 | |  | Idem |
| CPubmedP3 | Pubmed citationsP3 | | Numeric | 4 | 0-9000 | |  | Idem |
| CJournalP3 | Journal site citationsP3 | | Numeric | 4 | 0-9000 | |  | Idem |
| SocialP3 | Social media tweetsP3 | | Numeric | 4 | 0-9000 | |  | Idem |
| **Objective 4: Numbers of participants who became facilitators** | | | | | | | | |
| Facilit | Participant facilitated | | Numeric | 1 | 1  2 | Yes  No | |  |
| Factotal | Total modules facilitated | | Numeric | 2 | 0-99 |  | | Idem |
